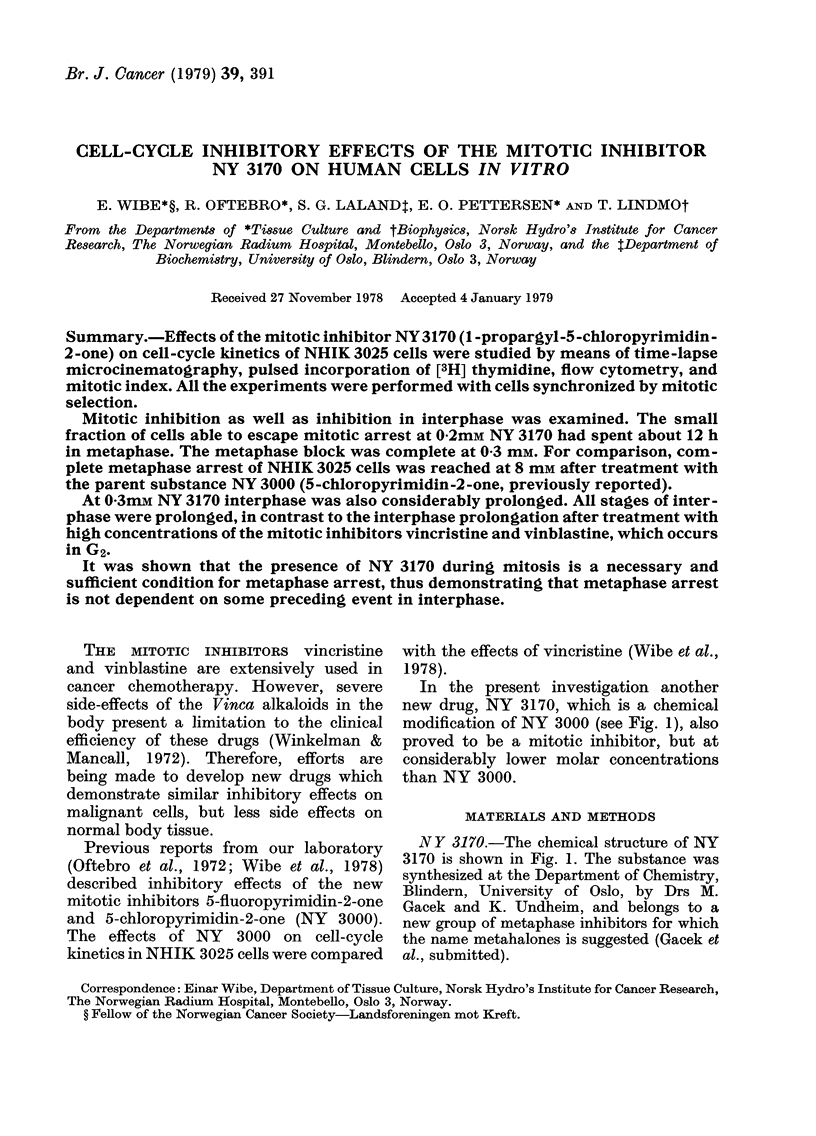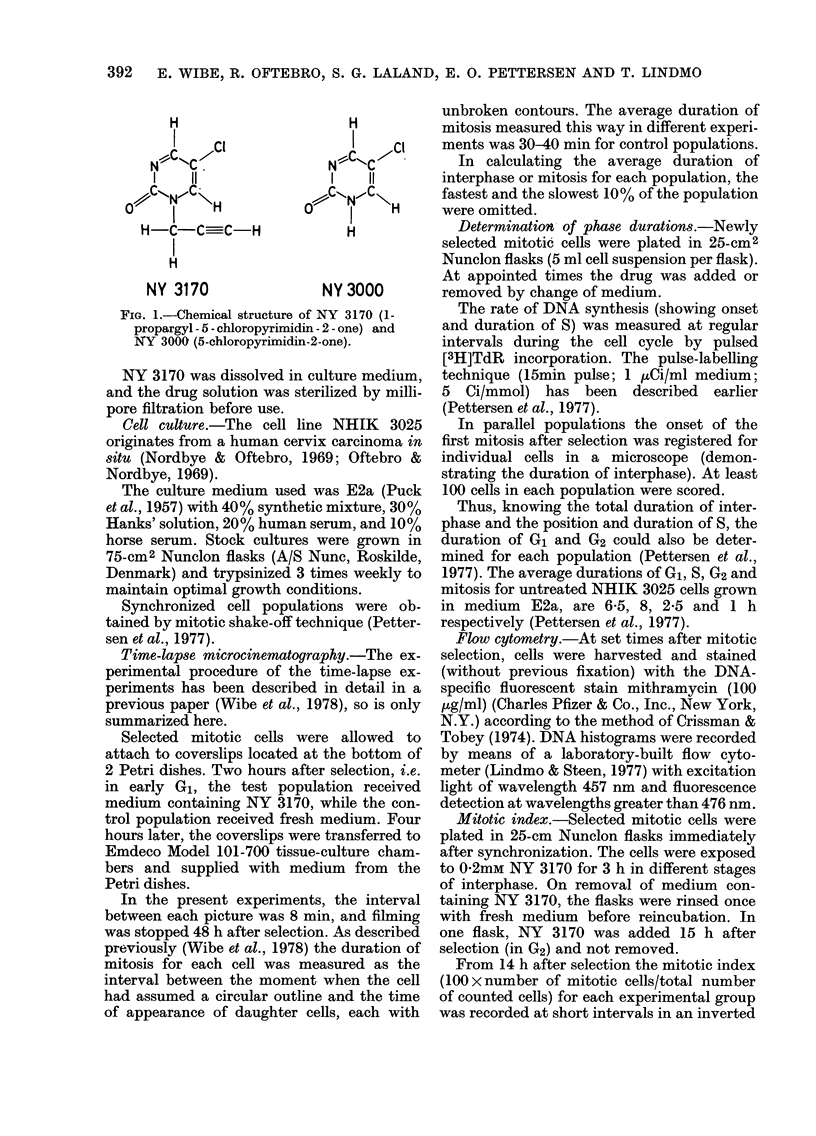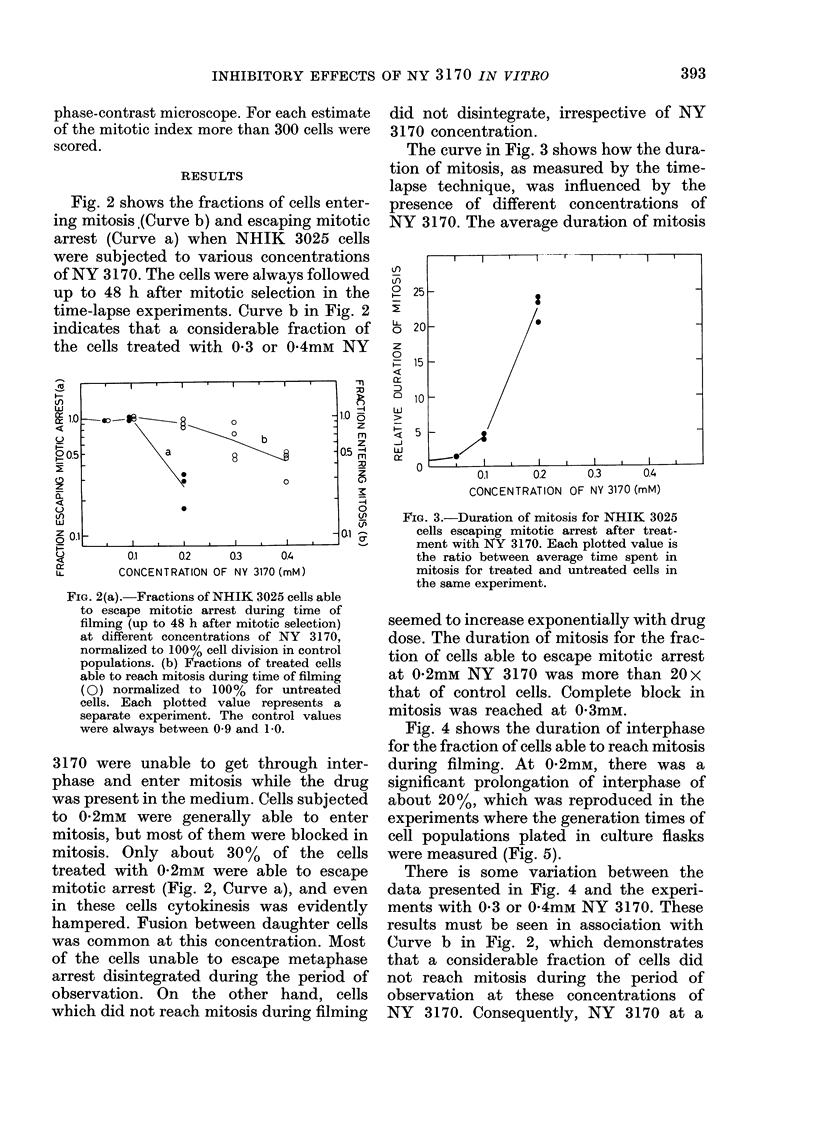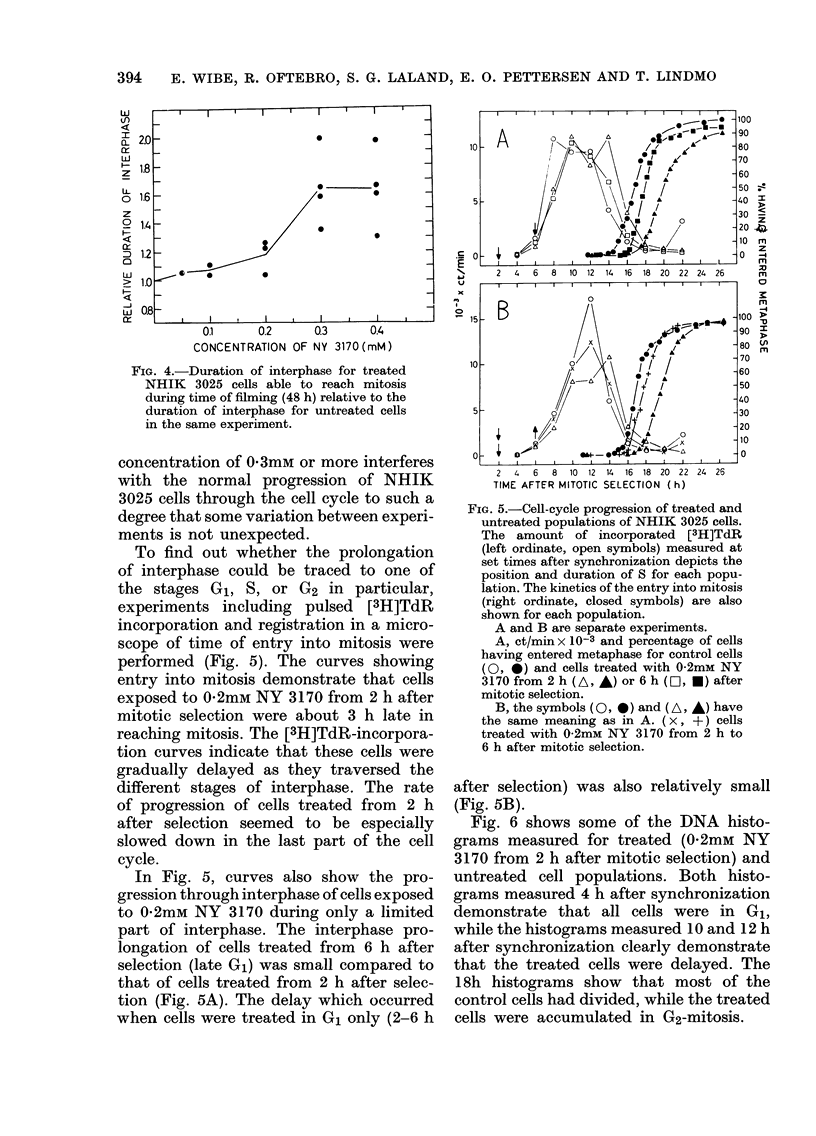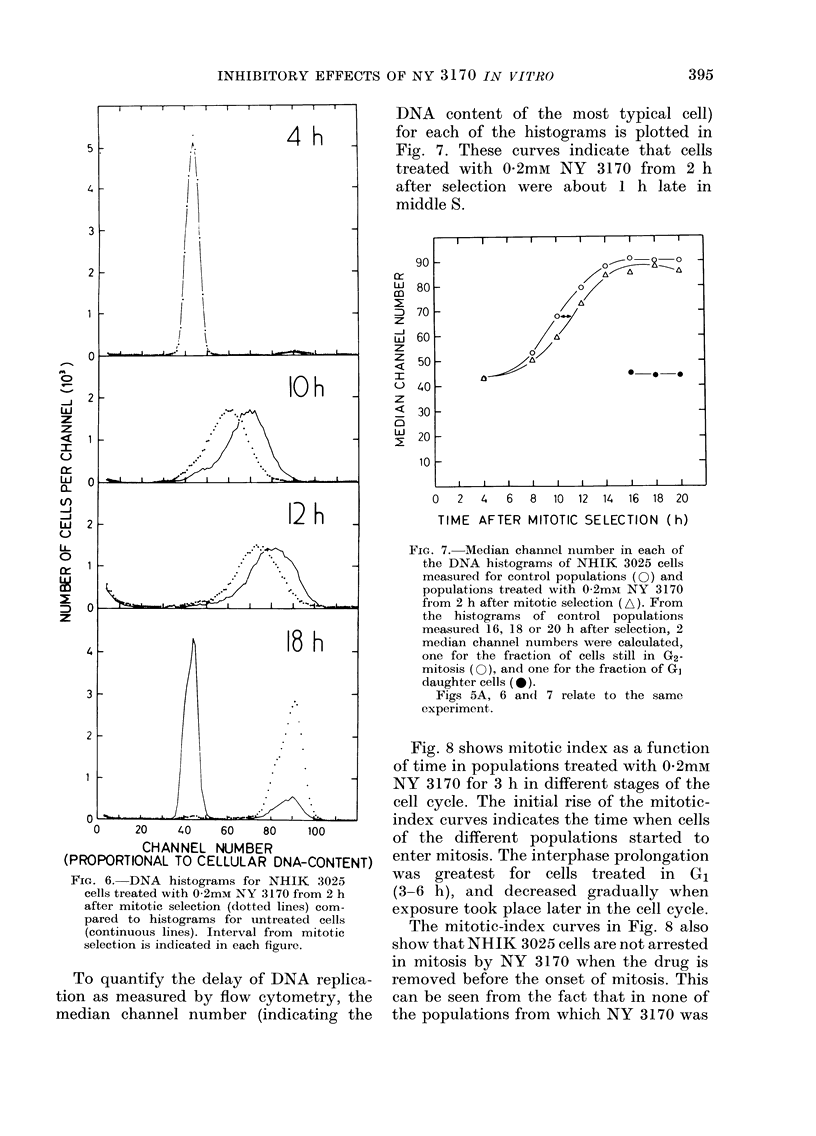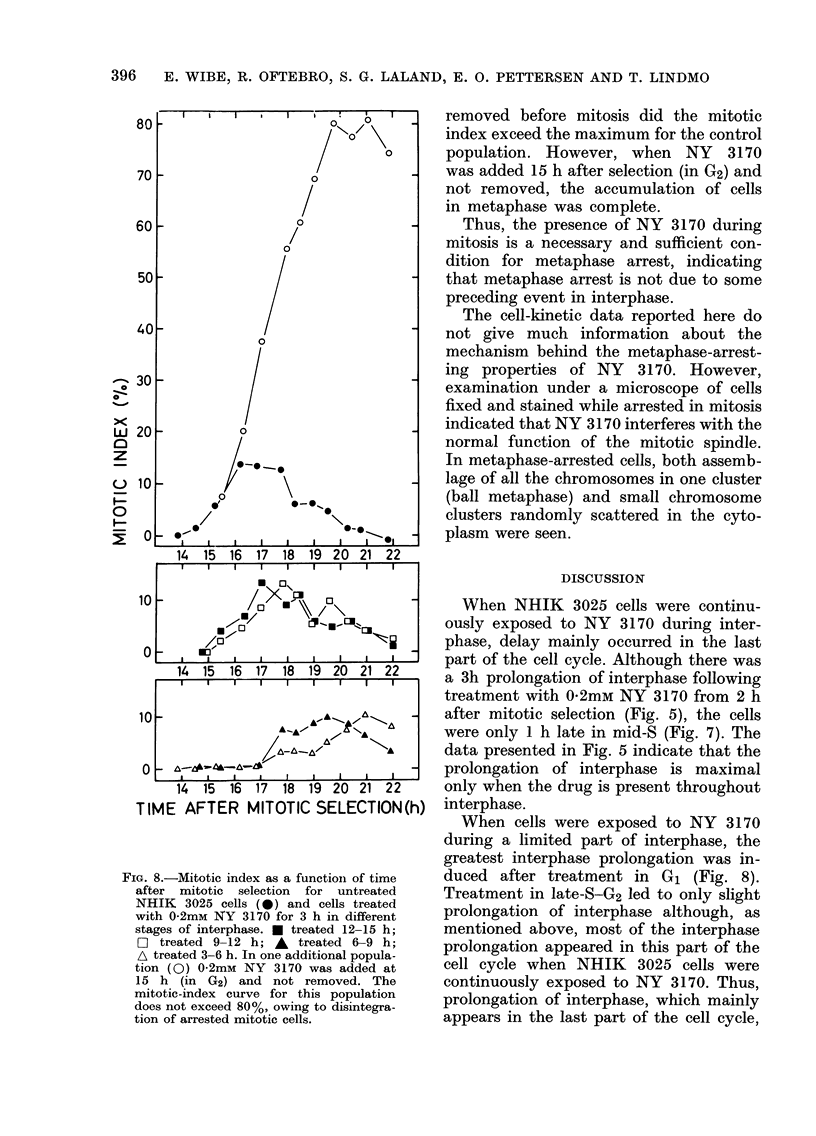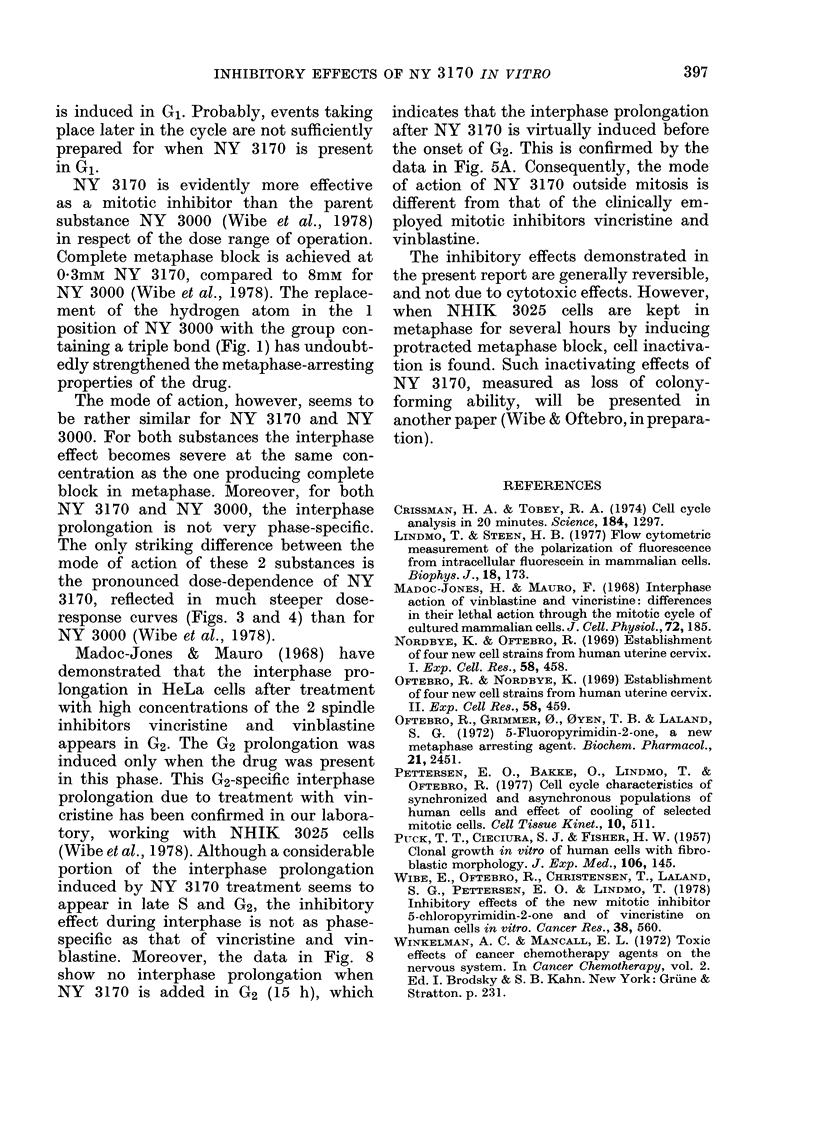# Cell-cycle inhibitory effects of the mitotic inhibitor NY 3170 on human cells in vitro.

**DOI:** 10.1038/bjc.1979.71

**Published:** 1979-04

**Authors:** E. Wibe, R. Oftebro, S. G. Laland, E. O. Pettersen, T. Lindmo

## Abstract

Effects of the mitotic inhibitor NY 3170 (1-propargyl-5-chloropyrimidin-2-one) on cell-cycle kinetics of NHIK 3025 cells were studied by means of time-lapse microcinematography, pulsed incorporation of [3H] thymidine, flow cytometry, and mitotic index. All the experiments were performed with cells synchronized by mitotic selection. Mitotic inhibition as well as inhibition in interphase was examined. The small fraction of cells able to escape mitotic arrest at 0.2mM NY 3170 had spent about 12 h in metaphase. The metaphase block was complete at 0.3 mM. For comparison, complete metaphase arrest of NHIK 3025 cells was reached at 8 mM after treatment with the parent substance NY 3000 (5-chloropyrimidin-2-one, previously reported). At 0.3mM NY 3170 interphase was also considerably prolonged. All stages of interphase were prolonged, in contrast to the interphase prolongation after treatment with high concentrations of the mitotic inhibitors vincristine and vinblastine, which occurs in G2. It was shown that the presence of NY 3170 during mitosis is a necessary and sufficient condition for metaphase arrest, thus demonstrating that metaphase arrest is not dependent on some preceding event in interphase.


*Br. J. Cancer* (1979) **39**, 391

### CELL-CYCLE INHIBITORY EFFECTS OF THE MITOTIC INHIBITOR NY 3170 ON HUMAN CELLS *IN VITRO*

E. WIBE*§, R. OFTEBRO*, S. G. LALAND‡, E. O. PETTERSEN* AND T. LINDMO†

*From the Departments of *Tissue Culture and †Biophysics, Norsk Hydro's Institute for Cancer Research, The Norwegian Radium Hospital, Montebello, Oslo 3, Norway, and the ‡Department of Biochemistry, University of Oslo, Blindern, Oslo 3, Norway*

Received 27 November 1978 Accepted 4 January 1979

**Summary.—Effects of the mitotic inhibitor NY 3170 (1-propargyl-5-chloropyrimidin-2-one) on cell-cycle kinetics of NHIK 3025 cells were studied by means of time-lapse microcinematography, pulsed incorporation of [$^3$H] thymidine, flow cytometry, and mitotic index. All the experiments were performed with cells synchronized by mitotic selection.**

**Mitotic inhibition as well as inhibition in interphase was examined. The small fraction of cells able to escape mitotic arrest at 0·2mM NY 3170 had spent about 12 h in metaphase. The metaphase block was complete at 0·3 mM. For comparison, complete metaphase arrest of NHIK 3025 cells was reached at 8 mM after treatment with the parent substance NY 3000 (5-chloropyrimidin-2-one, previously reported).**

**At 0·3mM NY 3170 interphase was also considerably prolonged. All stages of interphase were prolonged, in contrast to the interphase prolongation after treatment with high concentrations of the mitotic inhibitors vincristine and vinblastine, which occurs in $G_2$.**

**It was shown that the presence of NY 3170 during mitosis is a necessary and sufficient condition for metaphase arrest, thus demonstrating that metaphase arrest is not dependent on some preceding event in interphase.**

THE MITOTIC INHIBITORS vincristine and vinblastine are extensively used in cancer chemotherapy. However, severe side-effects of the *Vinca* alkaloids in the body present a limitation to the clinical efficiency of these drugs (Winkelman & Mancall, 1972). Therefore, efforts are being made to develop new drugs which demonstrate similar inhibitory effects on malignant cells, but less side effects on normal body tissue.

Previous reports from our laboratory (Oftebro *et al.*, 1972; Wibe *et al.*, 1978) described inhibitory effects of the new mitotic inhibitors 5-fluoropyrimidin-2-one and 5-chloropyrimidin-2-one (NY 3000). The effects of NY 3000 on cell-cycle kinetics in NHIK 3025 cells were compared with the effects of vincristine (Wibe *et al.*, 1978).

In the present investigation another new drug, NY 3170, which is a chemical modification of NY 3000 (see Fig. 1), also proved to be a mitotic inhibitor, but at considerably lower molar concentrations than NY 3000.

### MATERIALS AND METHODS

*NY 3170*.—The chemical structure of NY 3170 is shown in Fig. 1. The substance was synthesized at the Department of Chemistry, Blindern, University of Oslo, by Drs M. Gacek and K. Undheim, and belongs to a new group of metaphase inhibitors for which the name metahalones is suggested (Gacek *et al.*, submitted).

Correspondence: Einar Wibe, Department of Tissue Culture, Norsk Hydro's Institute for Cancer Research, The Norwegian Radium Hospital, Montebello, Oslo 3, Norway.

§ Fellow of the Norwegian Cancer Society—Landsforeningen mot Kreft.

FIG. 1.—Chemical structure of NY 3170 (1-propargyl-5-chloropyrimidin-2-one) and NY 3000 (5-chloropyrimidin-2-one).

NY 3170 was dissolved in culture medium, and the drug solution was sterilized by millipore filtration before use.

*Cell culture.*—The cell line NHIK 3025 originates from a human cervix carcinoma *in situ* (Nordbye & Oftebro, 1969; Oftebro & Nordbye, 1969).

The culture medium used was E2a (Puck *et al.*, 1957) with 40% synthetic mixture, 30% Hanks' solution, 20% human serum, and 10% horse serum. Stock cultures were grown in 75-cm$^2$ Nunclon flasks (A/S Nunc, Roskilde, Denmark) and trypsinized 3 times weekly to maintain optimal growth conditions.

Synchronized cell populations were obtained by mitotic shake-off technique (Pettersen *et al.*, 1977).

*Time-lapse microcinematography.*—The experimental procedure of the time-lapse experiments has been described in detail in a previous paper (Wibe *et al.*, 1978), so is only summarized here.

Selected mitotic cells were allowed to attach to coverslips located at the bottom of 2 Petri dishes. Two hours after selection, *i.e.* in early $G_1$, the test population received medium containing NY 3170, while the control population received fresh medium. Four hours later, the coverslips were transferred to Emdeco Model 101-700 tissue-culture chambers and supplied with medium from the Petri dishes.

In the present experiments, the interval between each picture was 8 min, and filming was stopped 48 h after selection. As described previously (Wibe *et al.*, 1978) the duration of mitosis for each cell was measured as the interval between the moment when the cell had assumed a circular outline and the time of appearance of daughter cells, each with unbroken contours. The average duration of mitosis measured this way in different experiments was 30–40 min for control populations.

In calculating the average duration of interphase or mitosis for each population, the fastest and the slowest 10% of the population were omitted.

*Determination of phase durations.*—Newly selected mitotic cells were plated in 25-cm$^2$ Nunclon flasks (5 ml cell suspension per flask). At appointed times the drug was added or removed by change of medium.

The rate of DNA synthesis (showing onset and duration of S) was measured at regular intervals during the cell cycle by pulsed [$^3$H]TdR incorporation. The pulse-labelling technique (15min pulse; 1 $\mu$Ci/ml medium; 5 Ci/mmol) has been described earlier (Pettersen *et al.*, 1977).

In parallel populations the onset of the first mitosis after selection was registered for individual cells in a microscope (demonstrating the duration of interphase). At least 100 cells in each population were scored.

Thus, knowing the total duration of interphase and the position and duration of S, the duration of $G_1$ and $G_2$ could also be determined for each population (Pettersen *et al.*, 1977). The average durations of $G_1$, S, $G_2$ and mitosis for untreated NHIK 3025 cells grown in medium E2a, are 6·5, 8, 2·5 and 1 h respectively (Pettersen *et al.*, 1977).

*Flow cytometry.*—At set times after mitotic selection, cells were harvested and stained (without previous fixation) with the DNA-specific fluorescent stain mithramycin (100 $\mu$g/ml) (Charles Pfizer & Co., Inc., New York, N.Y.) according to the method of Crissman & Tobey (1974). DNA histograms were recorded by means of a laboratory-built flow cytometer (Lindmo & Steen, 1977) with excitation light of wavelength 457 nm and fluorescence detection at wavelengths greater than 476 nm.

*Mitotic index.*—Selected mitotic cells were plated in 25-cm Nunclon flasks immediately after synchronization. The cells were exposed to 0·2mM NY 3170 for 3 h in different stages of interphase. On removal of medium containing NY 3170, the flasks were rinsed once with fresh medium before reincubation. In one flask, NY 3170 was added 15 h after selection (in $G_2$) and not removed.

From 14 h after selection the mitotic index (100 × number of mitotic cells/total number of counted cells) for each experimental group was recorded at short intervals in an inverted phase-contrast microscope. For each estimate of the mitotic index more than 300 cells were scored.

### RESULTS

Fig. 2 shows the fractions of cells entering mitosis (Curve b) and escaping mitotic arrest (Curve a) when NHIK 3025 cells were subjected to various concentrations of NY 3170. The cells were always followed up to 48 h after mitotic selection in the time-lapse experiments. Curve b in Fig. 2 indicates that a considerable fraction of the cells treated with 0·3 or 0·4mM NY

FIG. 2(a).—Fractions of NHIK 3025 cells able to escape mitotic arrest during time of filming (up to 48 h after mitotic selection) at different concentrations of NY 3170, normalized to 100% cell division in control populations. (b) Fractions of treated cells able to reach mitosis during time of filming (O) normalized to 100% for untreated cells. Each plotted value represents a separate experiment. The control values were always between 0·9 and 1·0.

3170 were unable to get through interphase and enter mitosis while the drug was present in the medium. Cells subjected to 0·2mM were generally able to enter mitosis, but most of them were blocked in mitosis. Only about 30% of the cells treated with 0·2mM were able to escape mitotic arrest (Fig. 2, Curve a), and even in these cells cytokinesis was evidently hampered. Fusion between daughter cells was common at this concentration. Most of the cells unable to escape metaphase arrest disintegrated during the period of observation. On the other hand, cells which did not reach mitosis during filming did not disintegrate, irrespective of NY 3170 concentration.

The curve in Fig. 3 shows how the duration of mitosis, as measured by the time-lapse technique, was influenced by the presence of different concentrations of NY 3170. The average duration of mitosis

FIG. 3.—Duration of mitosis for NHIK 3025 cells escaping mitotic arrest after treatment with NY 3170. Each plotted value is the ratio between average time spent in mitosis for treated and untreated cells in the same experiment.

seemed to increase exponentially with drug dose. The duration of mitosis for the fraction of cells able to escape mitotic arrest at 0·2mM NY 3170 was more than 20× that of control cells. Complete block in mitosis was reached at 0·3mM.

Fig. 4 shows the duration of interphase for the fraction of cells able to reach mitosis during filming. At 0·2mM, there was a significant prolongation of interphase of about 20%, which was reproduced in the experiments where the generation times of cell populations plated in culture flasks were measured (Fig. 5).

There is some variation between the data presented in Fig. 4 and the experiments with 0·3 or 0·4mM NY 3170. These results must be seen in association with Curve b in Fig. 2, which demonstrates that a considerable fraction of cells did not reach mitosis during the period of observation at these concentrations of NY 3170. Consequently, NY 3170 at a

FIG. 4.—Duration of interphase for treated NHIK 3025 cells able to reach mitosis during time of filming (48 h) relative to the duration of interphase for untreated cells in the same experiment.

concentration of 0·3mM or more interferes with the normal progression of NHIK 3025 cells through the cell cycle to such a degree that some variation between experiments is not unexpected.

To find out whether the prolongation of interphase could be traced to one of the stages $G_1$, S, or $G_2$ in particular, experiments including pulsed [$^3$H]TdR incorporation and registration in a microscope of time of entry into mitosis were performed (Fig. 5). The curves showing entry into mitosis demonstrate that cells exposed to 0·2mM NY 3170 from 2 h after mitotic selection were about 3 h late in reaching mitosis. The [$^3$H]TdR-incorporation curves indicate that these cells were gradually delayed as they traversed the different stages of interphase. The rate of progression of cells treated from 2 h after selection seemed to be especially slowed down in the last part of the cell cycle.

In Fig. 5, curves also show the progression through interphase of cells exposed to 0·2mM NY 3170 during only a limited part of interphase. The interphase prolongation of cells treated from 6 h after selection (late $G_1$) was small compared to that of cells treated from 2 h after selection (Fig. 5A). The delay which occurred when cells were treated in $G_1$ only (2–6 h

FIG. 5.—Cell-cycle progression of treated and untreated populations of NHIK 3025 cells. The amount of incorporated [$^3$H]TdR (left ordinate, open symbols) measured at set times after synchronization depicts the position and duration of S for each population. The kinetics of the entry into mitosis (right ordinate, closed symbols) are also shown for each population.

A and B are separate experiments.

A, ct/min × $10^{-3}$ and percentage of cells having entered metaphase for control cells (○, ●) and cells treated with 0·2mM NY 3170 from 2 h (△, ▲) or 6 h (□, ■) after mitotic selection.

B, the symbols (○, ●) and (△, ▲) have the same meaning as in A. (×, +) cells treated with 0·2mM NY 3170 from 2 h to 6 h after mitotic selection.

after selection) was also relatively small (Fig. 5B).

Fig. 6 shows some of the DNA histograms measured for treated (0·2mM NY 3170 from 2 h after mitotic selection) and untreated cell populations. Both histograms measured 4 h after synchronization demonstrate that all cells were in $G_1$, while the histograms measured 10 and 12 h after synchronization clearly demonstrate that the treated cells were delayed. The 18h histograms show that most of the control cells had divided, while the treated cells were accumulated in $G_2$-mitosis.

FIG. 6.—DNA histograms for NHIK 3025 cells treated with 0·2mM NY 3170 from 2 h after mitotic selection (dotted lines) compared to histograms for untreated cells (continuous lines). Interval from mitotic selection is indicated in each figure.

To quantify the delay of DNA replication as measured by flow cytometry, the median channel number (indicating the DNA content of the most typical cell) for each of the histograms is plotted in Fig. 7. These curves indicate that cells treated with 0·2mM NY 3170 from 2 h after selection were about 1 h late in middle S.

FIG. 7.—Median channel number in each of the DNA histograms of NHIK 3025 cells measured for control populations (○) and populations treated with 0·2mM NY 3170 from 2 h after mitotic selection (△). From the histograms of control populations measured 16, 18 or 20 h after selection, 2 median channel numbers were calculated, one for the fraction of cells still in $G_2$-mitosis (○), and one for the fraction of $G_1$ daughter cells (●).

Figs 5A, 6 and 7 relate to the same experiment.

Fig. 8 shows mitotic index as a function of time in populations treated with 0·2mM NY 3170 for 3 h in different stages of the cell cycle. The initial rise of the mitotic-index curves indicates the time when cells of the different populations started to enter mitosis. The interphase prolongation was greatest for cells treated in $G_1$ (3–6 h), and decreased gradually when exposure took place later in the cell cycle.

The mitotic-index curves in Fig. 8 also show that NHIK 3025 cells are not arrested in mitosis by NY 3170 when the drug is removed before the onset of mitosis. This can be seen from the fact that in none of the populations from which NY 3170 was

FIG. 8.—Mitotic index as a function of time after mitotic selection for untreated NHIK 3025 cells (●) and cells treated with 0·2mM NY 3170 for 3 h in different stages of interphase. ■ treated 12–15 h; □ treated 9–12 h; ▲ treated 6–9 h; △ treated 3–6 h. In one additional population (○) 0·2mM NY 3170 was added at 15 h (in $G_2$) and not removed. The mitotic-index curve for this population does not exceed 80%, owing to disintegration of arrested mitotic cells.

removed before mitosis did the mitotic index exceed the maximum for the control population. However, when NY 3170 was added 15 h after selection (in $G_2$) and not removed, the accumulation of cells in metaphase was complete.

Thus, the presence of NY 3170 during mitosis is a necessary and sufficient condition for metaphase arrest, indicating that metaphase arrest is not due to some preceding event in interphase.

The cell-kinetic data reported here do not give much information about the mechanism behind the metaphase-arresting properties of NY 3170. However, examination under a microscope of cells fixed and stained while arrested in mitosis indicated that NY 3170 interferes with the normal function of the mitotic spindle. In metaphase-arrested cells, both assemblage of all the chromosomes in one cluster (ball metaphase) and small chromosome clusters randomly scattered in the cytoplasm were seen.

### DISCUSSION

When NHIK 3025 cells were continuously exposed to NY 3170 during interphase, delay mainly occurred in the last part of the cell cycle. Although there was a 3h prolongation of interphase following treatment with 0·2mM NY 3170 from 2 h after mitotic selection (Fig. 5), the cells were only 1 h late in mid-S (Fig. 7). The data presented in Fig. 5 indicate that the prolongation of interphase is maximal only when the drug is present throughout interphase.

When cells were exposed to NY 3170 during a limited part of interphase, the greatest interphase prolongation was induced after treatment in $G_1$ (Fig. 8). Treatment in late-S–$G_2$ led to only slight prolongation of interphase although, as mentioned above, most of the interphase prolongation appeared in this part of the cell cycle when NHIK 3025 cells were continuously exposed to NY 3170. Thus, prolongation of interphase, which mainly appears in the last part of the cell cycle, is induced in $G_1$. Probably, events taking place later in the cycle are not sufficiently prepared for when NY 3170 is present in $G_1$.

NY 3170 is evidently more effective as a mitotic inhibitor than the parent substance NY 3000 (Wibe *et al.*, 1978) in respect of the dose range of operation. Complete metaphase block is achieved at 0·3mM NY 3170, compared to 8mM for NY 3000 (Wibe *et al.*, 1978). The replacement of the hydrogen atom in the 1 position of NY 3000 with the group containing a triple bond (Fig. 1) has undoubtedly strengthened the metaphase-arresting properties of the drug.

The mode of action, however, seems to be rather similar for NY 3170 and NY 3000. For both substances the interphase effect becomes severe at the same concentration as the one producing complete block in metaphase. Moreover, for both NY 3170 and NY 3000, the interphase prolongation is not very phase-specific. The only striking difference between the mode of action of these 2 substances is the pronounced dose-dependence of NY 3170, reflected in much steeper dose-response curves (Figs. 3 and 4) than for NY 3000 (Wibe *et al.*, 1978).

Madoc-Jones & Mauro (1968) have demonstrated that the interphase prolongation in HeLa cells after treatment with high concentrations of the 2 spindle inhibitors vincristine and vinblastine appears in $G_2$. The $G_2$ prolongation was induced only when the drug was present in this phase. This $G_2$-specific interphase prolongation due to treatment with vincristine has been confirmed in our laboratory, working with NHIK 3025 cells (Wibe *et al.*, 1978). Although a considerable portion of the interphase prolongation induced by NY 3170 treatment seems to appear in late S and $G_2$, the inhibitory effect during interphase is not as phase-specific as that of vincristine and vinblastine. Moreover, the data in Fig. 8 show no interphase prolongation when NY 3170 is added in $G_2$ (15 h), which indicates that the interphase prolongation after NY 3170 is virtually induced before the onset of $G_2$. This is confirmed by the data in Fig. 5A. Consequently, the mode of action of NY 3170 outside mitosis is different from that of the clinically employed mitotic inhibitors vincristine and vinblastine.

The inhibitory effects demonstrated in the present report are generally reversible, and not due to cytotoxic effects. However, when NHIK 3025 cells are kept in metaphase for several hours by inducing protracted metaphase block, cell inactivation is found. Such inactivating effects of NY 3170, measured as loss of colony-forming ability, will be presented in another paper (Wibe & Oftebro, in preparation).

### REFERENCES

Crissman, H. A. & Tobey, R. A. (1974) Cell cycle analysis in 20 minutes. *Science*, **184**, 1297.

Lindmo, T. & Steen, H. B. (1977) Flow cytometric measurement of the polarization of fluorescence from intracellular fluorescein in mammalian cells. *Biophys. J.*, **18**, 173.

Madoc-Jones, H. & Mauro, F. (1968) Interphase action of vinblastine and vincristine: differences in their lethal action through the mitotic cycle of cultured mammalian cells. *J. Cell. Physiol.*, **72**, 185.

Nordbye, K. & Oftebro, R. (1969) Establishment of four new cell strains from human uterine cervix. I. *Exp. Cell. Res.*, **58**, 458.

Oftebro, R. & Nordbye, K. (1969) Establishment of four new cell strains from human uterine cervix. II. *Exp. Cell Res.*, **58**, 459.

Oftebro, R., Grimmer, Ø., Øyen, T. B. & Laland, S. G. (1972) 5-Fluoropyrimidin-2-one, a new metaphase arresting agent. *Biochem. Pharmacol.*, **21**, 2451.

Pettersen, E. O., Bakke, O., Lindmo, T. & Oftebro, R. (1977) Cell cycle characteristics of synchronized and asynchronous populations of human cells and effect of cooling of selected mitotic cells. *Cell Tissue Kinet.*, **10**, 511.

Puck, T. T., Cieciura, S. J. & Fisher, H. W. (1957) Clonal growth *in vitro* of human cells with fibroblastic morphology. *J. Exp. Med.*, **106**, 145.

Wibe, E., Oftebro, R., Christensen, T., Laland, S. G., Pettersen, E. O. & Lindmo, T. (1978) Inhibitory effects of the new mitotic inhibitor 5-chloropyrimidin-2-one and of vincristine on human cells *in vitro*. *Cancer Res.*, **38**, 560.

Winkelman, A. C. & Mancall, E. L. (1972) Toxic effects of cancer chemotherapy agents on the nervous system. In *Cancer Chemotherapy*, vol. 2. Ed. I. Brodsky & S. B. Kahn. New York: Grüne & Stratton. p. 231.